# Influence of HLA on human partnership and sexual satisfaction

**DOI:** 10.1038/srep32550

**Published:** 2016-08-31

**Authors:** J. Kromer, T. Hummel, D. Pietrowski, A. S. Giani, J. Sauter, G. Ehninger, A. H. Schmidt, I. Croy

**Affiliations:** 1 Smell & Taste Clinic, Department of Otorhinolaryngology, Medizinische Fakultät Carl Gustav Carus, Technische Universität Dresden, Fetscherstr 74, 01307 Dresden, Germany; 2 DKMS German Bone Marrow Donor Center, Kressbach 1, 72072 Tübingen, Germany; 3 Department of Internal Medicine, Medizinische Fakultät Carl Gustav Carus, Technische Universität Dresden, Fetscherstr 74, 01307 Dresden, Germany; 4 Department of Psychotherapy and Psychosomatic Medicine, Medizinische Fakultät Carl Gustav Carus, Technische Universität Dresden, Fetscherstr 74, 01307 Dresden, Germany.

## Abstract

The major histocompatibility complex (MHC, called HLA in humans) is an important genetic component of the immune system. Fish, birds and mammals prefer mates with different genetic MHC code compared to their own, which they determine using olfactory cues. This preference increases the chances of high MHC variety in the offspring, leading to enhanced resilience against a variety of pathogens. Humans are also able to discriminate HLA related olfactory stimuli, however, it is debated whether this mechanism is of behavioural relevance. We show on a large sample (N = 508), with high-resolution typing of HLA class I/II, that HLA dissimilarity correlates with partnership, sexuality and enhances the desire to procreate. We conclude that HLA mediates mate behaviour in humans.

Body odours can create immediate sexual attraction. Both, men and women of various cultures seek out perfumes which highlight their body odour in the most desirable way. The famous novel “Perfume” by Patrick Suskind describes a perfume made of body odours that drives people into ecstasy and makes them forget civilized behaviour. However, recent research indicates that the ‘olfactory match’ between people, rather than a universally irresistible smell might be the key to olfactory attraction^1^. We investigated the relation of HLA dissimilarity, which is perceived through the olfactory chemosignaling, with human mate choice.

In animals, immunologic compatibility determines individual variation of mate choice and is partly conveyed by olfactory cues^2^,^3^,^4^. The individual genetic major histocompatibility complex (MHC) represents a part of the adaptive immune system. MHC molecules are located on the surface of cells and play a key role in discriminating between endogenous and exogenous, potentially pathogenic material. Genes of the MHC gene cluster are expressed in a co-dominant way. When given a choice, mice and sticklebacks prefer mates with dissimilar MHC molecules compared to their own^2^,^3^. This is advantageous from an evolutionary point of view: The co-dominant expression increases the chances of high MHC variety in the offspring of MHC dissimilar compared to MHC similar couples, which in turn leads to enhanced resilience amongst a variety of pathogens^5^.

MHC related mate choice is guided by the sense of smell in vertebrates. Parts of the heavy chains of the MHC glycoproteins are detected in various body fluids like saliva, urine and sweat^6^. In addition, it has been suggested that MHC molecules influence microorganisms of the skin which are involved in the formation of body odours^7^. The precise mechanisms by which those MHC molecules shape the body odor are still under discussion^8^. However, MHC determined odors activate vomeronasal and olfactory neurons^4^.

In humans, MHC is called human leucocyte antigen (HLA). HLA alleles are grouped into class I (encompassing amongst others HLA-A, HLA-B and HLA-C), class II (encompassing amongst others HLA-DR, HLA-DQ and HLA-DP) and class III alleles (e.g. HSP 70, TNF α and Factor B). HLA class I molecules are expressed by almost every nucleated cell in the body. Their function lies in the presentation of proteins to T cells. HLA class II molecules are located in cells of the immune system and show fragments from phagolysosomes to T cells^9^.

For a long time it has not been clear whether the mechanism of MHC related mate choice is evolutionarily conserved in humans. However, in 1995 it was shown that HLA similarity affects human body odour preference. In this study, 49 women preferred the body odour of HLA dissimilar men in comparison to similar men^10^. Preference of body odours of HLA dissimilar persons has been replicated in various studies^11^,^12^,^13^, however another study showed no impact of HLA on body odour preferences^14^ and it has been suggested that an intermediate level of HLA dissimilarity might be optimal in terms of body odour preference^15^.

Most of those studies examined only a few of the HLA loci (mainly -A, -B and -DR) at comparably low resolution (e.g. HLA subgroups) and for most studies, HLA similarity was no determined for each HLA loci, but a total score was analysed (for excellent overview compare^16^).

Taken together, those studies suggest that HLA compatibility may influence body odour preference such that dissimilar body odours are preferred over similar ones. Oral contraception may disrupt this effect^11^.

It is an interesting question, how humans are able to perceive HLA related peptides. Humans have no functional vomeronasal organ^17^,^18^, hence perception must be established through a different pathway. In fact, receptors expressed in the olfactory epithelium may be involved in this chemosensory communication^19^ and it has been shown recently that women are able to differentiate pure HLA related peptide ligands^1^. However, the mechanisms are not delineated yet (compare^20^,^21^ for ongoing debate).

In animals, immunologic compatibility is a major factor in mate choice^2^,^3^. In humans, studies show mixed effects^16^. A study from 1983 reports that married couples shared more HLAA and -B alleles that expected by chance^22^. However, the mixed ethnicities in this study may have caused this effect and after control of ethnicity, no effect of HLA was observed. Other studies report - in line with the animal results - that couples share fewer alleles than expected by chance^23^,^24^ and results are influenced by cultural background^22^,^25^. To our knowledge, only one study examined whether HLA related mate choice is behaviourally relevant in humans. The authors showed in 48 couples that partners who shared few HLA alleles were more satisfied with their sex life compared to partners who shared many HLA alleles^26^.

We investigated whether HLA similarity affects different aspects of partnership, such as general satisfaction, sexual satisfaction and the desire to procreate with the respective partner. Further, body odour attractiveness was examined. As it has been reported, that women prefer body odours of HLA heterozygote men before homozygote ones^12^, we additionally tested the impact of homo- and heterozygosity.

## Methods

The study was approved by the ethical board of the University of Dresden (EK 195062013) and was carried out in accordance with the approved guidelines. Written informed consent was obtained from all participants.

### Participants

Over a period of nine months, heterosexual couples were recruited at the University campus. Couples were only included if both partners were aged 18 years and older and had normal olfactory function as ascertained by use of the “Sniffn’Sicks” screening test^27^. In total, 254 couples were included. The group of women was aged between 18 and 60 years (mean: 24.3 years; s.d.: 5.6), male participants were aged between 18 and 55 years (mean: 26.14 years; s.d.: 6.0). The duration of partnership was 1 month to 32 years (mean: 4 years; s.d.: 57.9 months). Most of the couples (91%, n = 231) had no children. Four women were pregnant at the time of examination. Those four were excluded from the analysis. 52.3% (n = 132) of the couples shared a household together.

### Procedure

Participants were asked to provide a DNA sample using buccal swabs for HLA-typing. Afterwards, they filled out a questionnaire (compare Supplementary Information) including descriptive questions (age, duration of partnership, numbers of children, health status) and rating about the satisfaction with the partnership (“How satisfied are you with your partnership?”)) and with partnership sexuality (“How satisfied are you with the sexuality in your partnership?”) on a scale from 0 to 10 (“not satisfied at all” to “extremely satisfied”) and the attractiveness of their partners body odour (“How attractive is your partners body odour to you (without perfumes, deodorant etc.)?” 0 to 10 (“not attractive at all” to extremely attractive”). In addition, participants stated whether they wanted to have (more) children with their partner (yes/maybe/no). The experimenter instructed the partners to answer the questionnaires independently of each other and supervised this procedure.

In contrast to previous studies^10^,^11^,^12^,^13^,^14^,^15^,^25^,^26^,^28^ HLA analysis was based on high-resolution typing. The structure of the antigen recognition site (ARS) of the HLA molecule determines the recognition of foreign antigens^29^. Probably, similar mechanisms mediate the effect of HLA molecules on mating choice^1^,^30^. It is therefore essential, to take into account the exact structure of the ARS. The 2 field resolution provides the information required: Alleles that differ in the first two fields differ by one or more nucleotide substitutions that change the amino-acid sequence of the encoded ARS^31^.

DNA samples were analysed at the ASHI-accredited DKMS Life Science Lab (Dresden, Germany) using a workflow for high-throughput high resolution HLA-typing^32^. In particular, exons 2 and 3 of HLA-A, -B, -C, -DRB1, -DQB1 and -DPB1 were sequenced by next-generation sequencing (NGS). For 97.8% of all loci high-resolution results could be achieved. In 2.2% of the samples only an intermediate resolution could be achieved due to unresolved and potential exon shuffling.

To match HLA alleles between couples, typing results were analyzed at the level of the 2-field HLA subgroup (representing specific HLA proteins) and compared for each locus separately. Any two alleles that were identical on that level were defined as a match. In addition, non-identical alleles that fell within the same G-group were also defined as a match, since these alleles have identical nucleotide sequences across antigen recognition sites. To handle HLA-typing results with only intermediate resolution, we used the most probable HLA phenotype, based haplotype frequencies derived from a sample of 507,008 German individuals.

At each locus there could be either two (e.g. both HLA-typing results of that locus were found to be matching between a couple), one or no matches. The locus in question was categorized as being ‘similar’ in case of two matching HLA-alleles at the level of the 2-field HLA subgroup and ‘dissimilar’ else. Additionally, all participants were classified as homo- or heterozygous for each HLA locus. Heterozygosity of a participant was defined per HLA locus as a non-match between his or her two alleles at the level of the 2-field HLA subgroup.

### Statistical analysis

The statistical analysis was performed using SPSS (Statistical Package for the Social Sciences, version 19.0, SPSS Inc., Chicago, Illinois, USA).

Data were analysed with a general mixed model approach. The fixed effects of HLA class (2), HLA match (2) and the interaction on partnership satisfaction and partnership sexuality were examined. In the first step, each couple served as subject, sex (2) and HLA locus (6) as repeated measurement. For the 2.2% of the samples, where only an intermediate resolution could be achieved, the most probable typing was used and the individual probability of correct HLA typing was added as weighting coefficient in the analyses. Partnership duration was included as covariate in a second model. As the dependent variables were correlated (r = 0.58, p < 0.001), the respective other depended variable was added as random effect in addition to partnership duration in a third model. In the second step, the effect of sex was analysed. Therefore, only HLA class I alleles (3) (which showed significant effects in the first step) were included in the model and the main effect of HLA match (2) was examined, separately for men and women. Accordingly, each individual served as subject in this analysis. In the third step, analysis focused on individual HLA class I alleles. Therefore three mixed models were calculated per sex with HLA match (2) as fixed main effect and this data was Bonferroni-corrected (factor 3).

The non-parametric variable of longing for children was analysed using a Chi-square test. For this analysis, women aged older than 40 were excluded (N = 10). In the first step, the impact of HLA match (2) on longing for children (3) was tested separately for HLA class I and II. As class II revealed no significant results, further analysis focused on class I. Sex differences were examined in the second step. The impact of HLA match (2) on longing for children (3) was tested separately for men and women. As there were no significant effects in men, the last step focused on women. Here, the impact of HLA match (2) on longing for children (3) was tested separately for each HLA class I allele (3). This data was Bonferroni-corrected (factor 3). In a second model, participants, who already had children (N = 16) were excluded from the analysis.

The effects of HLA class (2) and HLA match (2) on body odour attraction were examined similar to the mixed model analysis performed for partnership satisfaction and partnership sexuality. As body odour attraction was correlated to partnership satisfaction (r = 0.46, p < 0.001) and partnership sexuality (r = 0.49, p < 0.001), a model including these variables as covariates was analysed.

The effect of homo- and heterozygosity on ratings was examined using a generalized mixed model approach, similar to the one above. In the first step, the fixed effects of HLA class (2), HLA homo-/heterozygosity (2) and the interaction were examined. As there were no significant effects, further statistical testing was abandoned. The level of significance (α-level) was set at p < 0.05 for all analyses.

## Results

### HLA class I dissimilarity relates to partnership and sexual satisfaction

Partnership and sexual satisfaction were significantly related to HLA class I match. For both, partnership and sexual satisfaction, there was a significant interaction effect of HLA class and HLA similarity (partnership satisfaction: F(249, 1) = 14.1, p < 0.001; sexual satisfaction F(249, 1) = 5.1, p = 0.023). HLA class I dissimilarity was significantly associated with higher partnership satisfaction (F(249, 1) = 18.7, p < 0.001) and higher sexual satisfaction (F(496, 2) = 6.9, p = 0.008), while no such effect was found for HLA class II. Sex specific analysis revealed that overall HLA class I dissimilarity was related to higher partnership satisfaction in women (F(1, 249) = 9.9 p = 0.002) as well as in men (men: F(1, 240) = 8.8, p = 0.003), while overall HLA class I dissimilarity was related to higher sexual satisfaction in women only (F(1, 240) = 7.9, p = 0.005).

**Figure 1:**
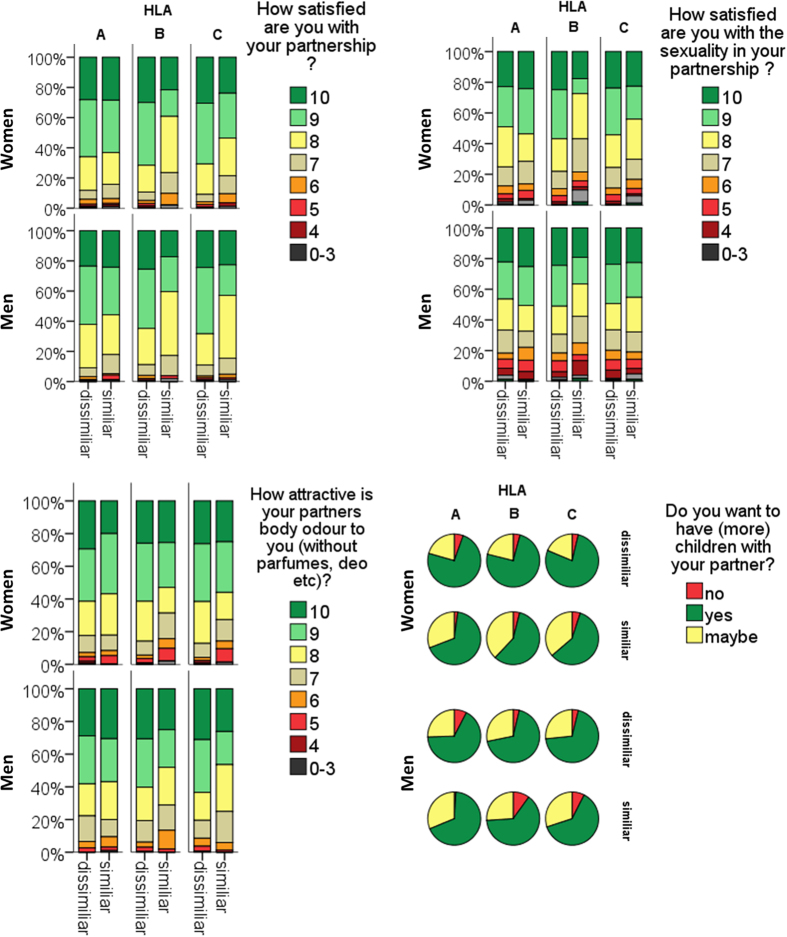
The impact of HLA similarity on partnership and sexual satisfaction, body odour attractiveness and the wish for children. In general, HLA class I (HLA,–C) dissimilar couples expressed higher satisfaction, body odour attractiveness and a stronger wish for children. HLA,B dissimilarity significantly related to sexual satisfaction in women: pcorr = 0.003 and men: pcorr = 0.027. HLA–C dissimilarity contributed to partnership satisfaction in men p corr = 0.033, in women also HLA–C p corr = 0.027 as well as HLA,B p corr = 0.003. No effect was observed for HLA,A and class II. Women with a HLA–C-dissimilar partner expressed higher longing for children pcorr = 0.027. The error bar indicates the standard mean error.

Sex and HLA type specific analysis revealed that sexual satisfaction in women was related to HLA-B dissimilarity (F(1, 240) = 14.5; p_corr_ < 0.001). Partnership satisfaction in women was related to HLA-B (F(1, 240) = 8.3; p_corr_ = 0.01) as well as HLA-C (F(1, 240) = 6.6; p_corr_ = 0.03). In men, higher partnership satisfaction was related to HLA-C dissimilarity (p_corr_ = 0.033, d = 0.25). Further, HLA-B dissimilarity was related to enhanced sexual satisfaction in men (p_corr_ = 0.027, d = 0.32). despite the lack of an overall HLA class I effect. No effect was observed for HLA-A (Fig. 1, Supplementary Table 1).

Control for relationship duration did not impact the results. The impact of HLA class I on partnership satisfaction was stable after control of partnership sexuality (F(249, 1) = 14.7, p < 0.001). However, HLA class I dissimilarity did not significantly increase sexual satisfaction, after control of partnership satisfaction.

### Women are more likely to want children with HLA class I dissimilar partners

HLA class I dissimilarity was significantly related to a positive answer to the question of whether participants wanted to have (more) children with their partner (Chi^2^ = 11.3, p = 0.003). No such effect was observed for HLA class II. However, while sex specific analysis revealed a preference of HLA dissimilarity in women (Chi^2^ = 16.6, p < 0.001), no such effect was observed for men. Within the group of women, dissimilarity in HLA class I allele B and C was related to the wish to have children with their partner, though after Bonferroni correction, only the impact of HLA-C remained significant (Chi^2^ = 8.9, p_corr_ = 0.03, compare Fig. 1 and Supplementary Table 1). Exclusion of participants who already had children, did not impact the results.

### HLA class I dissimilar partners are preferred in terms of body odor attractiveness

There was a significant interaction between the HLA similarity and HLA class (F(249, 1) = 6.3, p = 0.012), indicating that only HLA class I dissimilarity was significantly associated with higher body odor attractiveness (F(249, 1) = 8.2, p = 0.004). No such effect was observed for HLA class II (F(1, 1.5) = 0.4, p = 0.55). Sex specific analysis revealed, that HLA class I dissimilarity was related to women’s rating of partner body odor pleasantness (F(249, 1) = 4.2, p = 0.04, compare Fig. 1 and Supplementary Table 1), while no such effect was observed for men. Within the group of women, dissimilarity in HLA-B and -C class I alleles were related to higher body odor attractiveness rating of the respective partner, however those effects did not hold after Bonferroni correction.

There was no significant interaction between HLA similarity and HLA class (F(249, 1) = 0.1, p = 0.8) on body odor attraction after control for relationship duration and partnership satisfaction.

### HLA-heterozygote people are not preferred over HLA-homozygote

Heterozygosity was more frequent than homozygosity for each of the HLA alleles (compare Supplementary Table 2). However sexual and partnership satisfaction, were independent of whether people were in a relationship with HLA heterozygote or homozygote partners. Similarly, no significant effects were obtained for the partner’s body odor attractiveness. Further, the desire for children was not affected by homozygote or heterozygote partners.

## Discussion

The current investigation revealed that immunologic compatibility matters in terms of (1) partnership satisfaction, (2) sexual satisfaction, and (3) the wish to have children. Hence, subjects were generally most satisfied with their relationship if their partner exhibited a dissimilar HLA type. This effect was only evident for HLA alleles of class I, while no effect was found for class II alleles.

This supports and extends work from previous studies showing that humans are able to discriminate intranasally administered HLA-related peptides^1^ and prefer the body odour of HLA dissimilar individuals^10^,^11^,^12^,^28^. These results are also in line with studies showing that immunologic compatibility relates to mate choice in mammals^3^,^33^ and humans^23^,^24^,^25^. However, some authors report no preference of HLA dissimilarity in humans^14^,^15^. Differences in methodology may explain some of the variation: We examined established couples and used questionnaires while others examined body odour ratings of strangers. We further analysed the whole range of HLA class I and II alleles at comparably high resolution, while previous studies focused on few alleles. One has to keep in mind, that partnership satisfaction and partnership sexuality were correlated to each other thus whilst the impact of HLA class I on partnership satisfaction remained stable after correction of partnership sexuality, the converse did not. Further, our participants were rather satisfied with their partnership and partnership sexuality. This is in accordance to results from a representative German survey, where the vast majority of men stated to be somewhat to very satisfied with their partnership and sexuality^34^. Women were not included in this survey.

In our study, both sexes were significantly more attracted by their partner’s body odour if the couple had dissimilar HLA class I alleles. However, self-report body odour attractiveness ratings were influenced by partnership satisfaction and further studies are warranted where participants actually smell unmarked samples of their partner’s body odor. In line with our results, studies performed on strangers show that women prefer the body odour of HLA dissimilar compared to similar men^10^,^11^,^12^,^13^.

All beneficial effects of HLA dissimilarity were present in men and women. But as we examined couples, sex specific analyses have to be treated with caution. When a man is unsatisfied with his relationship, it may be due to HLA similarity or due to being engaged with a woman who is unsatisfied. In general, HLA similarity affected women more than men, especially in terms of sexual satisfaction and the wish to have children. In line with this female sexual dissatisfaction is related to HLA similarity^26^. We speculate that the bigger investment of women in offspring leads to a stronger, but most likely subconscious, emphasis on factors contributing to a healthy offspring.

HLA alleles contributed differently; class I alleles were found to be of much stronger importance than class II alleles. Especially HLA-B and - to somewhat lesser extent – HLA-C dissimilarity was related to enhanced partnership and sexual satisfaction and to the wish for children. Interestingly, it was found in birds, that MHC class I (corresponding to HLA, in humans), mainly -B, is pivotal for parasitic resistance^35^. However, in fish^36^ and mice^37^, diversity of MHC class II relates as well to parasite load and a conclusive explanation for why HLA class I was related to mate behaviour in humans while HLA class II was not, is lacking. The correlational approach of our study does not provide sufficient explanation here. Furthermore this correlational approach limits the interpretation: We assume, that HLA match between couples impacts partnership and sexual satisfaction as well as the wish for children and odour attractiveness rating. However, it is also possible, that the coherence is determined by an underlying third variable such as linkage effects due to non-HLA loci.

In contrast to HLA similarity, HLA heterozygosity was unrelated to any of the examined aspects of partnership satisfaction. Heterozygosity might be related to enhanced resistance against pathogens compared to homozygosity in humans^38^,^39^ and a similar mechanisms has been reported in mice^5^. However in a population of ox, goat and sheep no strong indications for an advantage of heterozygosity were found^40^ and it has been argued that homozygosity does not preclude health, but makes an individual and a population more vulnerable to infectious disease at a time of exposure^41^.

Heterozygote individuals hence may be preferred as mate partners, because they can better take care of the offspring^5^. In line, women prefer faces of heterozygote men^42^,^43^ and there is one study showing that body odour attractiveness ratings are higher for T-Shirts worn by HLA-B heterozygous compared to HLA-B homozygous donators^12^. However no preference of heterozygote individuals in term of partnership satisfaction, sexual satisfaction, the wish to procreate, or body odour rating was found in our study. In contrast to previous studies, high resolution HLA analysis performed here included the level of the 2-field HLA subgroup and hence was more sensitive to dissimilarity. Compared to 1-field analyses, this approach enhanced the chance for heterozygosity on the individual level and for non-match between partners on the partner level.

In conclusion, attraction is a miracle to most of us and only some of the many factors mediating mate choice involve odours. However, within the world of human olfaction, there seems to be no perfect mate but a perfect partner and this depends on HLA match.

## Additional Information

**How to cite this article**: Kromer, J. *et al.* Influence of HLA on human partnership and sexual satisfaction. *Sci. Rep.* **6**, 32550; doi: 10.1038/srep32550 (2016).

## Supplementary Material

Supplementary Information
