# Supplementary material for: Influence of HLA on human partnership and sexual satisfaction
Source: Sci Rep. 2016 Aug 31;6:32550. doi: 10.1038/srep32550 (PMC5006172; doi:10.1038/srep32550)
Supplement: Supplementary Information [file srep32550-s1.doc]

# Supplementary Information

**Influence of HLA on human partnership and sexual satisfaction**

Kromer J^1^, Hummel T^1^, Pietrowski D^1^, Giani AS^4^, Sauter J^4^, Ehninger G^3^, Schmidt AH^4^, Croy I^1,2 *^

1 Smell & Taste Clinic, Department of Otorhinolaryngology, 2 Department of Psychosomatic Medicine, 3 Department of Internal Medicine, TU Dresden, Germany; 4 DKMS German Bone Marrow Donor Center, Tübingen, Germany;

## Supplementary Methods

## Questionnaire

Code

*sex* □male □female *age* __years

*highest education:*

□ no graduation

□ 8th/9th grade

□ 10th grade

□ A-level

□ university degree

*Age of partner* __years

*Duration of partnership* ___ months or ___ years

*Do you and your partner live together?* □ yes □ no

*Do you have children together with your partner?* □ yes □ no

*Number of children __*

*Age of children ____________*

*Do you have children from another partner?* □ yes □ no

*Number of children __*

*Age of children ____________*

*Do you want to have (more) children with your partner ?*  □ yes □maybe □no

*How attractive is your partners body odor to you (without perfumes, deo etc.)?*

| 0 | 1 | 2 | 3 | 4 | 5 | 6 | 7 | 8 | 9 | 10 |
| --- | --- | --- | --- | --- | --- | --- | --- | --- | --- | --- |
| Not attractive at all | |  |  |  |  |  |  |  | Extremely attractive | |

How satisfied are you with your partnership?

| 0 | 1 | 2 | 3 | 4 | 5 | 6 | 7 | 8 | 9 | 10 |
| --- | --- | --- | --- | --- | --- | --- | --- | --- | --- | --- |
| Not satisfied at all | |  |  |  |  |  |  |  | Extremely satisfied | |

*How satisfied are you with your sexual relationship with your partner*?

| 0 | 1 | 2 | 3 | 4 | 5 | 6 | 7 | 8 | 9 | 10 |
| --- | --- | --- | --- | --- | --- | --- | --- | --- | --- | --- |
| Not satisfied at all | |  |  |  |  |  |  |  | Extremely satisfied | |

## Supplementary Results

|  | **HLA of partner** | **Partner-ship satis-faction** | **p; effect size d** | **Sexual satis-faction** | **p; effect size d** | **Body odor attractiveness** | **p; effect size d** | **Longing for children** | | | **p; effect size V** |
| --- | --- | --- | --- | --- | --- | --- | --- | --- | --- | --- | --- |
|  |  | **Mean± SD** |  | **Mean± SD** |  | **Mean± SD** |  | **yes** | **May-be** | **no** |  |
| **Class I** |  |  |  |  |  |  |  |  |  |  |  |
| ***HLA-A*** | |  |  |  |  |  |  |  |  |  |  |
| Men | similar N=95 | 8.47±1.49 | 0.18 | 7.97±1.98 | 0.76 | 8.52±1.47 | 0.82 | 63 | 29 | 2 | 0.02 |
|  | dissimilar N=153 | 8.7±1.12 |  | 7.98±1.96 |  | 8.56±1.3 |  | 97 | 37 | 18 |  |
| Women | similar N=95 | 8.63±1.36 | 0.61 | 8.19±1.73 | 0.93 | 8.45±1.3 | 0.45 | 63 | 29 | 3 | 0.04; 0.12 |
|  | dissimilar N=153 | 8.72±1.25 |  | 8.21±1.63 |  | 8.59±1.41 |  | 108 | 30 | 15 |  |
| ***HLA-B*** | |  |  |  |  |  |  |  |  |  |  |
| Men | similar N=51 | 8.29±1.29 | 0.04; 0.32 | **7.52±2.08** | **<0.01; 0.23** | 8.29±1.41 | 0.14 | 32 | 13 | 7 | 0.29 |
|  | dissimilar N=197 | 8.7±1.26 |  | **8.03±1.92** |  | 8.61±1.35 |  | 128 | 53 | 13 |  |
| Women | similar N=51 | **8.22±1.4** | **<0.01; 0.45** | **7.41±2.08** | **<0.01, 0.56** | 8.18±1.71 | 0.04;0.30 | 29 | 19 | 4 | 0.04; 0.11 |
|  | dissimilar N=197 | **8.81±1.24** |  | **8.41±1.48** |  | 8.63±1.25 |  | 142 | 40 | 14 |  |
| ***HLA-C*** | |  |  |  |  |  |  |  |  |  |  |
| Men | similar N=84 | **8.4±1.25** | **0.01; 0.25** | 7.88±1.98 | 0.84 | 8.4±1.26 | 0.28 | 50 | 24 | 9 | 0.41 |
|  | dissimilar N=164 | **8.72±1.28** |  | 7.74±1.96 |  | 8.61±1.42 |  | 110 | 42 | 11 |  |
| Women | similar N=84 | **8.38±1.47** | **0.01; 0.35** | 7.95±1.91 | 0.10 | 8.27±1.62 | 0.04; 0.28 | **47** | **29** | **7** | **0.01; 0.14** |
|  | dissimilar N=164 | **8.84±1.17** |  | 8.33±1.51 |  | 8.67±1.19 |  | **124** | **30** | **11** |  |
| **Class II** | |  |  |  |  |  |  |  |  |  |  |
| ***HLA-DR*** | |  |  |  |  |  |  |  |  |  |  |
| Men | similar N=59 | 8.63±1.31 | 0.43 | 8.1±1.98 | 0.42 | 8.59±1.49 | 0.73 | 41 | 14 | 4 | 0.71 |
|  | dissimilar N=189 | 8.58±1.26 |  | 7.86±1.96 |  | 8.52±1.33 |  | 119 | 52 | 16 |  |
| Women | similar N=59 | 8.63±1.53 | 0.69 | 8.34±1.94 | 0.47 | 8.58±1.47 | 0.80 | 41 | 14 | 4 | 0.99 |
|  | dissimilar N=189 | 8.7±1.22 |  | 8.14±1.58 |  | 8.52±1.34 |  | 130 | 45 | 14 |  |
| ***HLA-DQ*** | |  |  |  |  |  |  |  |  |  |  |
| Men | similar N=180 | 8.68±1.24 | 0.60 | 7.98±1.88 | 0.76 | 8.64±1.37 | 0.44 | 56 | 20 | 4 | 0.37 |
|  | dissimilar N=168 | 8.58±1.29 |  | 7.89±1.01 |  | 8.49±1.37 |  | 104 | 46 | 16 |  |
| Women | similar N=180 | 8.76±1.39 | 0.52 | 8.4±1.66 | 0.20 | 8.55±1.35 | 0.91 | 60 | 15 | 5 | 0.36 |
|  | dissimilar N=168 | 8.65±1.25 |  | 8.11±1.67 |  | 8.53±1.38 |  | 111 | 44 | 13 |  |
| **HLA- DP** | |  |  |  |  |  |  |  |  |  |  |
| Men | similar N=125 | 8.68±1.16 | 0.34 | 7.84±2.01 | 0.53 | 8.53±1.25 | 0.92 | 77 | 38 | 7 | 0.17 |
|  | dissimilar N=123 | 8.55±1.38 |  | 8.0±1.92 |  | 8.55±1.48 |  | 83 | 28 | 13 |  |
| Women | similar N=125 | 8.66±1.42 | 0.80 | 8.11±1.81 | 0.40 | 8.55±1.46 | 0.86 | 85 | 28 | 12 | 0.34 |
|  | dissimilar N=123 | 8.71±1.16 |  | 8.29±1.52 |  | 8.52±1.26 |  | 86 | 31 | 6 |  |

***Tab. S1: Ratings of partnership and sexual satisfaction, body odor attractiveness and wish for children in relation to HLA similarity. Significant (uncorrected) p-values of similar-dissimiliar comparisons are displayed. Results that hold for bonferroni correction are highlighted in bold. Effect sizes (Cohens d or Cramers V, respectively) are reported for significant results.***

|  |  | **Rating of** |  | **Mean± SD** | **Mean± SD** | **Mean± SD** | **yes** | **maybe** | **no** |
| --- | --- | --- | --- | --- | --- | --- | --- | --- | --- |
| Class I | HLA-A | women | homozygote (n=36) | 8.33± 1.77 | 7.5± 2.54 | 8.31± 1.8 | 26 | 7 | 3 |
|  |  |  | heterozygote (n=214) | 8.65± 1.17 | 7.99± 1.84 | 8.57± 1.29 | 147 | 52 | 15 |
|  |  | men | homozygote (n=27) | 8.44± 1.05 | 7.56± 1.89 | 8.07± 1.24 | 16 | 10 | 1 |
|  |  |  | heterozygote (n=216) | 8.72± 1.33 | 8.3± 1.62 | 8.6± 1.37 | 138 | 55 | 18 |
|  | HLA-B | women | homozygote (n=14) | 8.71± 0.91 | 7.29± 2.4 | 8.79± 1.65 | 13 | 2 | 0 |
|  |  |  | heterozygote (n=236) | 8.6± 1.29 | 7.95± 1.93 | 8.51± 1.34 | 160 | 57 | 18 |
|  |  | men | homozygote (n=12) | 8.42± 1.31 | 8.25± 1.77 | 8.58± 1.17 | 5 | 5 | 2 |
|  |  |  | heterozygote (n=231) | 8.7± 1.3 | 8.21± 1.67 | 8.54± 1.38 | 149 | 60 | 17 |
|  | HLA-C | women | homozygote (n=23) | 8.65± 1.27 | 7.48± 2.17 | 8.57± 1.47 | 15 | 6 | 2 |
|  |  |  | heterozygote (n=227) | 8.6± 1.28 | 7.96± 1.93 | 8.52± 1.36 | 158 | 53 | 16 |
|  |  | men | homozygote (n=29) | 8.69± 1.42 | 8.14± 1.98 | 8.66± 1.4 | 18 | 7 | 4 |
|  |  |  | heterozygote (n=214) | 8.69± 1.29 | 7.22± 1.63 | 8.53± 1.37 | 136 | 15 | 58 |
| Class II | HLA-DR | women | homozygote (n=26) | 8.46± 1.21 | 8.77± 0.91 | 8.04± 1.59 | 17 | 8 | 1 |
|  |  |  | heterozygote (n=224) | 8.45± 1.39 | 8.59± 1.31 | 7.9± 2.0 | 156 | 51 | 17 |
|  |  |  | homozygote (n=19) | 8.42± 1.54 | 8.68± 1.29 | 8.1± 1.91 | 14 | 2 | 3 |
|  | | | heterozygote (n=224) | 8.55± 1.36 | 8.69± 1.3 | 8.22± 1.65 | 140 | 63 | 16 |
|  | HLA-DQ | women | homozygote (n=36) | 8.58± 1.34 | 9.06± 0.86 | 8.14± 1.76 | 24 | 11 | 1 |
|  |  |  | heterozygote (n=214) | 8.52± 1.38 | 8.53± 1.32 | 7.88± 1.99 | 149 | 48 | 17 |
|  |  | men | homozygote (n=26) | 8.35± 1.39 | 8.73± 1.19 | 8.19± 1.74 | 18 | 3 | 5 |
|  |  |  | heterozygote (n=217) | 8.57± 1.34 | 8.68± 1.31 | 8.22± 1.66 | 136 | 60 | 16 |
|  | HLA-DP | women | homozygote (n=68) | 8.37± 1.17 | 8.62± 1.23 | 8.03± 1.75 | 41 | 22 | 5 |
|  |  |  | heterozygote (n=182) | 8.59± 1.44 | 8.6± 1.29 | 7.87± 2.03 | 132 | 37 | 13 |
|  |  | men | homozygote (n=45) | 8.53± 1.18 | 8.68± 1.15 | 8.02± 1.55 | 26 | 15 | 5 |
|  |  |  | heterozygote (n=198) | 8.55± 1.41 | 8.69± 1.33 | 8.26± 1.66 | 128 | 50 | 15 |

***Tab.S 2: Ratings of partnership and sexual satisfaction, body odour attractiveness and wish for children in relation to HLA status of the partner.***
